# Psychological Factors Predict Response to a Low Fermentable Oligo‐, di‐, Monosaccharide and Polyol Dietary Intervention in Irritable Bowel Syndrome: A Prospective Cohort Study

**DOI:** 10.1002/ueg2.70204

**Published:** 2026-03-24

**Authors:** Lauren P. Manning, Maaike Van Den Houte, Caroline J. Tuck, Lukas Van Oudenhove, Jessica R. Biesiekierski

**Affiliations:** ^1^ Department of Food Nutrition and Dietetics La Trobe University Melbourne Australia; ^2^ Laboratory for Brain‐Gut Axis Studies (LaBGAS) Translational Research Centre for Gastrointestinal Disorders Department of Chronic Diseases & Metabolism KU Leuven Leuven Belgium; ^3^ Rehabilitation Research Centre (REVAL) Hasselt University Diepenbeek Belgium; ^4^ Department of Allied Health Swinburne University Melbourne Australia; ^5^ Department of Psychological and Brain Sciences Dartmouth College Hanover New Hampshire USA; ^6^ Human Nutrition Group School of Agriculture Food and Ecosystem Sciences Faculty of Science University of Melbourne Melbourne Australia

**Keywords:** gastrointestinal symptoms, irritable bowel syndrome, low FODMAP diet, psychological predictors, quality of life

## Abstract

**Background:**

The low fermentable oligo‐, di‐, monosaccharide and polyol (FODMAP) diet (LFD) effectively manages irritable bowel syndrome (IBS), but predictors of treatment response remain unknown.

**Objective:**

This study investigated whether psychological factors predict symptom improvement and quality of life (QoL) outcomes following a LFD intervention.

**Methods:**

Adults with Rome IV‐defined IBS underwent a three‐phase LFD over 6 months. Primary outcomes were IBS symptom severity and QoL. Validated questionnaires assessed depressive, gastrointestinal‐specific anxiety (GSA), and somatic symptoms, illness perceptions, and treatment expectations. Latent class growth analysis (LCGA) and cross‐lagged panel models (CLPM) were used to identify symptom trajectories and examine directional relationships between psychological factors and outcomes, respectively.

**Results:**

112 participants (89% female, median age 30 ± 17 years) completed the study. LCGA identified distinct IBS symptom severity and QoL trajectories during the LFD. Higher baseline treatment credibility and expectancy predicted favourable symptom improvements but were unrelated to membership in the QoL trajectory. Elevated GSA, psychological distress (depression, anxiety, stress), and negative illness perceptions increased the likelihood of poorer outcomes. CLPM revealed that lower GSA and higher personal control preceded subsequent symptom reductions. Higher treatment expectancy predicted improved QoL and symptom outcomes over time, while QoL improvements reduced stress and GSA.

**Conclusion:**

Lower baseline GSA anxiety and higher treatment expectations consistently predict better response to all phases of the LFD. These findings will help clinicians identify optimal candidates for dietary intervention versus alternative treatments.

## Key Summary

- Summarise the established knowledge on this subject
  ◦ The low FODMAP diet benefits many with IBS, but non‐response is common.
  ◦ IBS frequently involves psychological distress, which is linked to greater gastrointestinal symptom severity.
  ◦ Expectancy, credibility and illness perceptions influence outcomes in other chronic conditions but are understudied in dietary IBS management.
  ◦ Longitudinal, phase‐specific interactions between psychological factors, symptoms and quality of life during the 3‐phase low FODMAP diet have not previously been examined.
- What are the significant and/or new findings of this study?
  ◦ Higher baseline expectancy, credibility, personal control and more positive illness perceptions predicted more favourable symptom and quality of life trajectories.
  ◦ Gastrointestinal‐specific anxiety strongly predicted poorer symptom response; reductions in gastrointestinal‐specific anxiety preceded later symptom improvement.
  ◦ Expectancy showed bidirectional effects with symptoms and quality of life, while quality of life improvements preceded reductions in stress and gastrointestinal‐specific anxiety.
  ◦ Brief psychological screening (using tools such as VSI, CEQ, IPQ‐R) may guide personalised treatment selection between dietary and psychological therapies.

## Graphical abstract

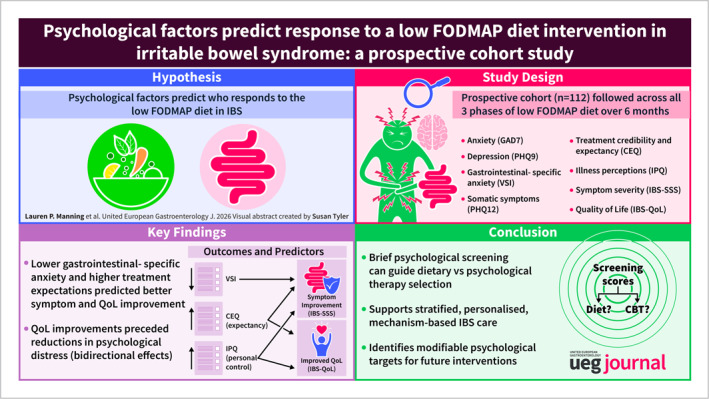

## 1 Introduction

Irritable bowel syndrome (IBS) is a chronic disorder of gut‐brain interaction (DGBI), affecting approximately 4.1% of the global population [1, 2]. Recurrent abdominal pain and altered bowel habits substantially impair quality of life (QoL) [3]. Psychological comorbidity is common, with meta‐analyses confirming elevated rates of depressive and anxiety symptoms in IBS patients [4, 5] with psychological distress correlating with increased gastrointestinal symptom severity [6, 7]. Effective strategies that target both symptoms [8, 9, 10, 11] and psychosocial contributors are therefore crucial.

The low fermentable oligosaccharide, disaccharide, monosaccharide, and polyol (FODMAP) diet (LFD) is an evidence‐based structured dietary approach that improves symptoms for many people with IBS [12, 13, 14], although 20%–50% do not respond [15] and many discontinue the protocol. Understanding which patients benefit most remains a major clinical need [16].

Psychological factors influence symptom perception and adherence [9]. Yet, their role in dietary treatment response remains underexplored. Prior work suggests that higher psychological distress predicts less favourable outcomes [10, 11]. However, these studies have not examined dynamic relationships (between gastrointestinal symptoms, QoL, and psychological symptoms) over time and often focus on general anxiety rather than *gastrointestinal‐specific* anxiety (GSA) and rarely evaluate *cognitive* predictors such as expectations or illness perceptions.

Cognitive factors such as treatment expectancy, treatment credibility, and illness perceptions are well‐established predictors of outcomes across chronic symptom conditions and are likely relevant to treatment response in IBS [17, 18, 19, 20, 21, 22, 23]. Expectancy and credibility reflect how convincing and personally beneficial patients perceive an intervention to be [18], while illness perceptions shape how individuals interpret and manage their condition [22]. These cognitive factors influence treatment engagement, coping, and symptom reporting across chronic conditions [17, 18, 19, 20, 21, 22, 23]. Despite their established importance, they have not been investigated as predictors of response to the LFD in IBS. Given the heterogeneity of IBS in psychological comorbidity, symptom presentation, and treatment response patterns, understanding how cognitive factors shape dietary outcomes is particularly important.

To address these gaps, this clinical trial aimed to assess psychological and cognitive predictors of response to a three‐phase LFD in IBS. Objective 1 was to determine whether baseline (1) treatment credibility and treatment expectancy, (2) illness perceptions, and (3) psychological symptoms (depression, general anxiety, GSA, somatic symptom severity, and stress) predicted changes in gastrointestinal symptoms and QoL improvement across the LFD. Objective 2 was to examine dynamic, directional relationships between changes in these psychological factors and clinical outcomes over time.

## 2 Methods

### 2.1 Study Design

**FIGURE 1:**
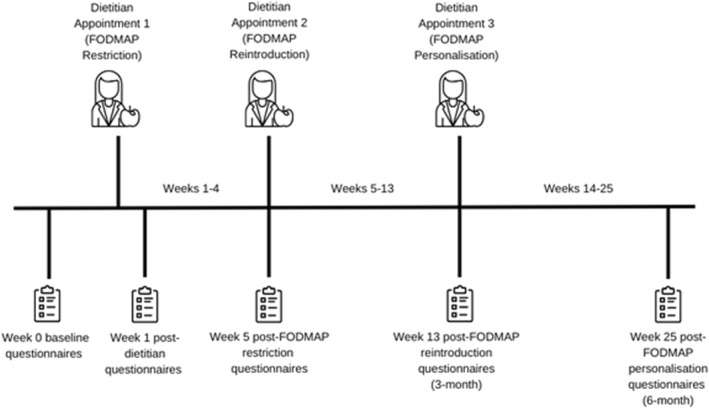
Timeline of participant appointments and data collection.

This study utilised a prospective, uncontrolled longitudinal cohort design to evaluate clinical and psychological changes during routine delivery of the LFD. Participants were recruited between August 2018 and April 2022. Dietitian consultations occurred at week 1 (Phase 1: Restriction), week 5 (Phase 2: Reintroduction), and 3 months (Phase 3: Personalisation). Questionnaires were completed at 5 time points over a 6‐month period: pre‐dietitian/baseline (week 0), post‐dietitian (week 1), and then at 4 weeks, three months, and 6 months after the initial dietitian appointment (weeks 5, 13, and 25, respectively) (Figure 1). Consultations initially took place in‐person and later transitioned online due to COVID‐19 restrictions, with most consultations ultimately occurring virtually.

### 2.2 Participants

Adults who had not yet seen a dietitian for IBS management, met the Rome IV criteria for IBS, displayed good English literacy skills, and were able to provide informed consent were eligible. Participants were included if medications or psychological therapies were stable for > 12 weeks. Exclusion criteria were: pregnancy; gastrointestinal conditions other than IBS (e.g., inflammatory bowel disease, celiac disease, or dyspepsia), probiotic use, and age < 18 years. Recruitment occurred through dietetic clinics within Melbourne, Australia, social media, and an online low‐FODMAP product retailer (FodShop).

### 2.3 Measures

#### 2.3.1 Demographic Variables

Information on participants' age, gender, education, employment, country of birth, ethnicity, geographic location, medication use, and complementary therapy use was collected at baseline, week 5, 3 and 6 months.

#### 2.3.2 Psychological and Cognitive Measures (Predictors)

The Credibility and Expectancy Questionnaire (CEQ) [24] assessed treatment credibility and treatment expectancy. Visceral Sensitivity Index (VSI) [25, 26] assessed GSA. Illness perceptions were measured using the Illness Perception Questionnaire‐Revised (IPQ‐R) [27]. The Patient Health Questionnaire‐9 (PHQ‐9) [28], Generalised Anxiety Disorder 7‐Item Scale (GAD‐7) [29], and PHQ‐12 [30] assessed depressive, anxiety, and somatic symptom severity, respectively. The Perceived Stress Scale (PSS) [31] measures psychological stress.

#### 2.3.3 Outcome Measures

Symptom severity (IBS Symptom Severity Score; IBS‐SSS) [32] and quality of life (IBS Quality of Life scale; IBS‐QoL) [33, 34] were the primary outcomes. Full questionnaire details are provided in the Supporting Information S1: Materials S1.

### 2.4 Treatment ‐ The Low FODMAP Diet

Participants received up to three consultations with dietitians trained in the LFD following established guidelines [16, 35]. After each appointment, participants received discount codes for access to low‐FODMAP speciality products to support adherence. Further intervention details are in the Supporting Information S1: Materials S2.

### 2.5 Bias

Dietitians were blinded to the questionnaires and participant responses to avoid influencing participant reporting. Standardised education materials and a consultation structure were used to minimise variability in dietary counselling.

### 2.6 Statistical Analysis

Analysis was performed using Statistical Analysis System (SAS) version 9.4 [36]. Demographic data are presented using mean ± standard deviation (SD) for normally distributed data or median and interquartile range (IQR) for non‐normally distributed data.

#### 2.6.1 Objective 1: Baseline Predictors of Response

To investigate whether baseline cognitive factors and psychological symptoms were related to response patterns (symptoms and QoL) to the LFD, latent class growth analysis (LCGA) was used. LGCA identifies distinct response trajectory classes by clustering combinations of intercepts (values at baseline) and (linear and higher order) slopes (the rate and direction of change) [37, 38]. Baseline psychological and cognitive variables were used to predict class membership to symptom and QoL response trajectories. Details of the LCGA modelling are outlined in the Supporting Information S1: Materials S3.

#### 2.6.2 Objective 2: Dynamic Relationships Over Time

First, linear mixed models (LMM) analysis was used to test whether psychological and cognitive predictor variables changed over time. If there was a significant change in a variable indicated by significant linear, quadratic or cubic effects of time it was used in a subsequent analysis to determine the directional relationship between that variable and gastrointestinal and QoL symptoms.

**FIGURE 2:**
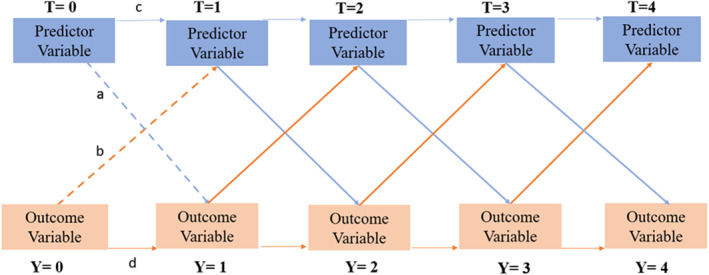
A cross‐lagged panel model showing the predictive pathways between the independent and dependent variables at each time point. Pathway (a) shows the predictive pathway between the predictor variable (*T* = 0) and the outcome variable (*Y* = 1); pathway (b) shows the predictive pathway between the outcome variable (*Y* = 0) and the predictor variable (*T* = 1); pathway (c) shows if the predictor variable at *T* = 0 predicts the score at *T* = 1; pathway (d) shows if the outcome variable at *Y* = 0 predicts the outcome variable score at *Y* = 1.

Cross‐lagged panel models (CLPM) were used to evaluate directional relationships between cognitive factors and psychological symptoms (X) and outcomes (gastrointestinal symptoms or QoL; Y) across consecutive time points [39]. CLPMs estimated whether X at time T predicted Y at time *T* + 1, and vice versa (Figure 2). Details of the development of the CLPMs are outlined in S3.

### 2.7 Sample Size

A priori sample size calculation was undertaken during study planning. The calculation was informed by published data examining treatment expectancy and symptom improvement [40], using the reported between group difference (placebo intervention vs. placebo intervention with meaningful healthcare practitioner relationship) and variability at the 3‐week time point to estimate the detectable effect size. Based on these parameters, a minimum of 48 participants was required to achieve 80% power (two‐sided independent samples *t*‐test), and the recruitment target was increased to 65 participants to achieve 90% power, allowing for potential attrition.

### 2.8 Missing Data

All analyses used all available participant data, including those who did not complete all follow‐up assessments. CLPMs used full‐information maximum likelihood (FIML), which accommodates missing data without listwise deletion. LCGAs used maximum likelihood estimation, allowing participants with incomplete follow‐up to contribute to model estimation.

### 2.9 Ethics

Ethics approval was provided by the La Trobe Human Ethics Council (HEC18260) on August 3^rd^, 2018. The trial was registered with the Australian New Zealand Clinical Trials Registry (ACTRN12624000514505). Written informed consent was obtained from all participants.

## 3 Results

### 3.1 Participants

**FIGURE 3:**
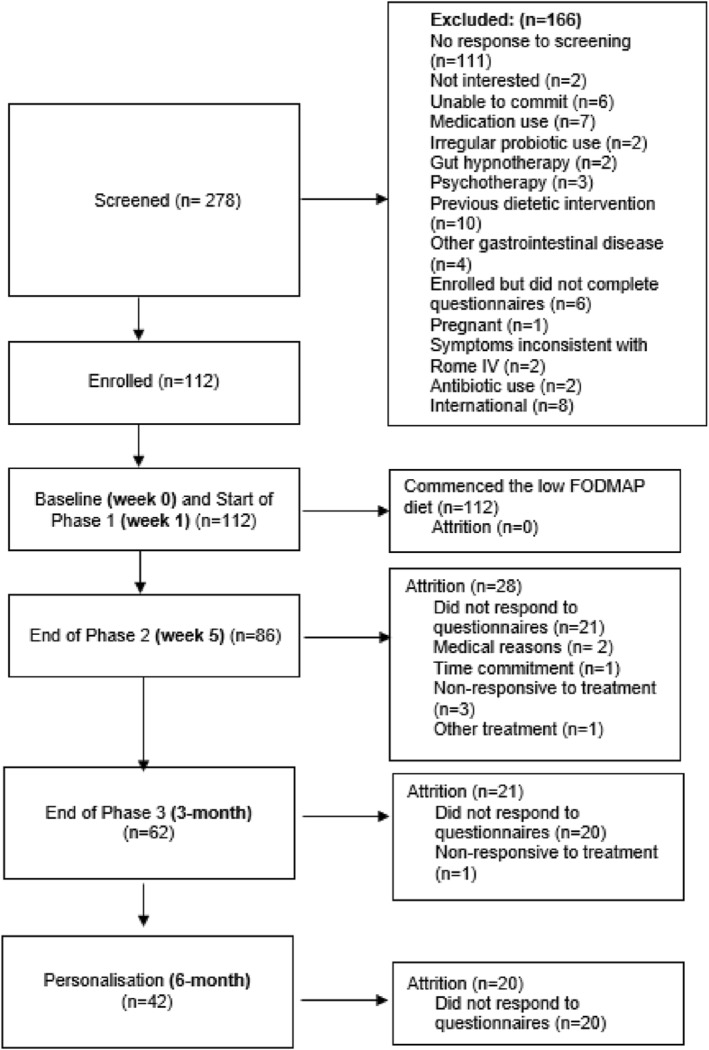
Participant flow diagram of screening, enrolment and attrition at each time point.

Of 278 individuals screened, 112 met inclusion criteria and enrolled (median age 30.5 ± 17 years, range 18–73; 89% female). All completed their initial dietitian visit (week 1). Retention declined over time, with 75% (*n* = 84) at week 5, 54% (*n* = 60) at 3 months, and 38% (*n* = 42) at 6 month (Figure 3). Baseline demographics are presented in Table 1.

**TABLE 1:** Baseline participant characteristics.

Age (median ± IQR)	30.5 ± 17.0 (18–73)
Gender, *n* (%)	
Female	100 (89%)
Male	11 (10%)
Not stated	1 (1%)
Education, *n* (%)	
No schooling	2 (2%)
Secondary school	11 (10%)
Diploma	17 (15%)
Higher education	82 (73%)
Geographic location, *n* (%)	
Metropolitan	91 (81%)
Regional	15 (14%)
Rural	6 (6%)
Work, *n* (%)	
Unemployed	11 (10%)
Casual	16 (14%)
Part‐time	24 (21%)
Full‐time	57 (51%)
Retired	4 (4%)
Baseline psychological variables (mean ± SD or median ± IQR^a^, range)	
CEQ‐credibility^a^	19.0 ± 6.0 (8–26)
CEQ‐expectancy^a^	20.0 ± 5.0 (9–27)
PHQ‐9^a^	6.5 ± 6 (0–22)
GAD‐7^a^	5 ± 5 (0–20)
PHQ‐12	8 ± 3 (0–18)
PSS	16 ± 7 (1–34)
VSI	49 ± 15 (8–75)
IBS‐SSS	277 ± 74 (25–492)
IBS QoL	59 ± 19 (13–99)
IPQ‐R identity^a^	4 ± 3 (1–12)
IPQ‐R time (acute/chronic)^a^	24 ± 4 (6–30)
IPQ‐R consequences	18 ± 5 (6–30)
IPQ‐R personal control^a^	23 ± 5 (5–30)
IPQ‐R treatment control^a^	19 ± 3 (5–25)
IPQ‐R illness coherence	14 ± 5 (5–25)
IPQ‐R time (cyclical)	15 ± 3 (4–20)
IPQ‐R emotional representations	19 ± 5 (6–30)

^a^ IQR: Interquartile range; CEQ: Credibility and Expectancy Questionnaire; PHQ‐9: Patient Health Questionnaire 9; GAD: Generalised Anxiety Disorder Scale 7; PHQ: Patient Health Questionnaire 12; PSS: Perceived Stress Scale; VSI: Visceral Sensitivity Index; IBS‐SSS: Irritable Bowel Syndrome Symptom Severity Score; IBS QoL: Irritable Bowel Syndrome Quality of Life; IPQ: Illness Perception Questionnaire.

### 3.2 Objective 1: Predictors of Response (LCGA)

#### 3.2.1 IBS Symptom Severity

**FIGURE 4:**
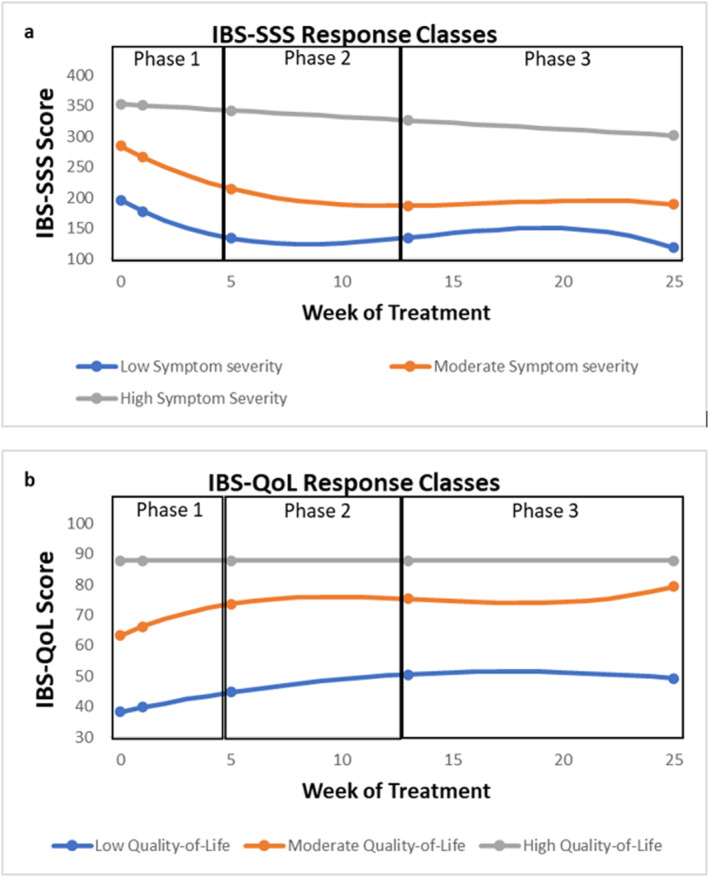
Estimated symptom trajectories based on the intercept and slopes of the LCGA solutions for (a) symptom severity and (b) quality of life. IBS‐SSS: Irritable Bowel Syndrome Symptom Severity Score; IBS‐QoL: Irritable Bowel Syndrome Quality of Life; LCGA: Latent Class Growth Analysis.

LCGA identified three distinct IBS‐SSS trajectories (Supporting Information S2: Table 1). Class 1 (22.5%) and class 2 (56.2%) showed steep initial symptom reductions during Phase 1, followed by more gradual changes in later phases (Figure 4a). Classes 1 and 2 differed only in baseline symptom severity: 196 ± 10.1 for class 1 (hereafter called *improvement ‐ low symptom severity class*) and 284 ± 17.6 for class 2 (hereafter called *improvement ‐ moderate symptom severity class*). Class 3 (21.3%) was characterised by very high baseline severity (352 ± 8.7) with minimal improvement (hereafter called *non‐improvement class*).

The *improvement ‐ low symptom severity class* served as the reference for predictor analyses (see Table 2). Compared to the improvement‐low class, higher baseline treatment credibility, and expectancy predicted a lower likelihood of belonging to the other two trajectory classes. More negative illness perceptions ‐ including stronger symptom attribution, longer perceived illness duration, lower treatment control, greater perceived consequences, and stronger emotional representations ‐ increased the likelihood of belonging to a higher baseline symptom severity class (all *p* < 0.05). Higher baseline depressive symptoms, GSA, somatic symptoms, and perceived stress also increased the likelihood of allocation to a higher baseline symptom severity class (all *p* < 0.05). Elevated generalised anxiety predicted membership in the non‐improvement class only (*p* = 0.0031).

**TABLE 2:** Predictive symptom class allocation per baseline treatment expectancy and credibility, psychological symptoms and illness perception questionnaire scores at baseline.

Class membership	Reference class: Improvement ‐ low symptom severity	
	Estimate (ß) + SE	*p*
CEQ ‐ credibility		
Improvement ‐ moderate symptom severity	−0.240 ± 0.098	0.014
Non‐improvement	−0.216 ± 0.104	0.016
CEQ ‐ expectancy		
Improvement ‐ moderate symptom severity	−0.182 ± 0.089	0.040
Non‐improvement	−0.178 ± 0.099	0.073
VSI		
Improvement ‐ moderate symptom severity	0.073 ± 0.024	0.002
Non‐improvement	0.171 ± 0.038	< 0.0001
PHQ‐9		
Improvement ‐ moderate symptom severity	0.252 ± 0.096	0.009
Non‐improvement	0.354 ± 0.106	0.0009
GAD‐7		
Improvement ‐ moderate symptom severity	0.132 ± 0.087	0.13
Non‐improvement	0.296 ± 0.100	0.003
PHQ‐12		
Improvement ‐ moderate symptom severity	0.200 ± 0.088	0.023
Non‐improvement	0.361 ± 0.108	0.0009
PSS		
Improvement ‐ moderate symptom severity	0.128 ± 0.050	0.010
Non‐improvement	0.157 ± 0.059	0.008
IPQ‐R identity		
Improvement ‐ moderate symptom severity	0.312 ± 0.170	0.067
Non‐improvement	0.613 ± 0.188	0.001
IPQ‐R time (acute/chronic)		
Improvement ‐ moderate symptom severity	0.214 ± 0.075	0.005
Non‐improvement	0.306 ± 0.099	0.002
IPQ‐R personal control		
Improvement ‐ moderate symptom severity	−0.098 ± 0.074	0.19
Non‐improvement	−0.150 ± 0.08	0.074
IPQ‐R ‐ treatment control		
Improvement ‐ moderate symptom severity	−0.226 ± 0.101	0.010
Non‐improvement	−0.271 ± 0.115	0.019
IPQ‐R‐ illness coherence		
Improvement ‐ moderate symptom severity	−0.095 ± 0.056	0.093
Non‐improvement	−0.064 ± 0.065	0.32
IPQ‐R‐ consequences		
Improvement ‐ moderate symptom severity	0.212 ± 0.080	0.008
Non‐improvement	0.375 ± 0.102	0.0003
IPQ‐R—Time (cyclical)		
Improvement ‐ moderate symptom severity	0.071 ± 0.088	0.42
Non‐improvement	−0.008 ± 0.098	0.93
IPQ‐R‐ emotional representations		
Improvement ‐ moderate symptom severity	0.159 ± 0.074	0.031
Non‐improvement	0.300 ± 0.090	0.0009

Abbreviations: CEQ: Credibility and Expectancy Questionnaire; GAD: Generalised Anxiety Disorder Scale 7; IPQ: Illness Perception Questionnaire; PHQ: Patient Health Questionnaire 12; PHQ‐9: Patient Health Questionnaire 9; PSS: Perceived Stress Scale; VSI: Visceral Sensitivity Index.

#### 3.2.2 Quality of Life

LCGA identified three distinct QoL trajectories (Supporting Information S2: Table 2): Class C (17%) maintained consistently high QoL throughout the trial (*high QoL)*. Class A (30.6%) began with low QoL and improved steadily with a slowing rate of change in later phases of the diet (*improvement—low QoL*), while Class B (52.4%) showed moderate baseline QoL with a steep early increase, temporary stabilisation during Phase 2, and renewed improvement after Phase 3 (*improvement ‐ moderate QoL*, Figure 4b).

The high QoL class served as the reference group when exploring baseline predictors of QoL class allocation (see Table 3). Baseline treatment credibility and expectancy were unrelated to QoL class allocation. Lower baseline illness coherence, personal control and treatment control predicted membership in the *improvement* ‐ *low QoL* class relative to the *high QoL* class (all *p* < 0.05). More negative illness perceptions ‐ specifically higher baseline scores on the timeline, consequences, and emotional representation domains ‐ and higher baseline psychological symptom severity (scores on the PHQ‐9, GAD‐7, VSI, PHQ‐12, and PSS) increased the likelihood of belonging to the *improvement* ‐ *low QoL* and the *improvement ‐ moderate QoL* classes relative to the *high QoL* class (all *p* < 0.05).

**TABLE 3:** Predictive quality of life class allocation per baseline treatment expectancy and credibility, psychological symptoms and illness perception questionnaire scores at week zero.

Class membership	Reference class ‐ high QoL	
	Estimate (ß) + SE	*p*
CEQ‐ credibility		
Improvement ‐ low QoL	−0.113 ± 0.852	0.18
Improvement ‐ moderate QoL	−0.063 ± 0.084	0.45
CEQ‐expectancy		
Improvement ‐ low QoL	−0.082 ± 0.089	0.36
Improvement ‐ moderate QoL	−0.033 ± 0.090	0.72
VSI		
Improvement ‐ low QoL	0.325 ± 0.080	0.0001
Improvement ‐ moderate QoL	0.228 ± 0.074	0.002
PHQ‐9		
Improvement ‐ low QoL	0.685 ± 0.209	0.001
Improvement ‐ moderate QoL	0.604 ± 0.203	0.003
GAD‐7		
Improvement ‐ low QoL	0.509 ± 0.133	0.0002
Improvement ‐ moderate QoL	0.460 ± 0.131	0.0005
PHQ‐12		
Improvement ‐ low QoL	0.310 ± 0.099	0.002
Improvement ‐ moderate QoL	0.193 ± 0.090	0.033
PSS		
Improvement ‐ low QoL	0.282 ± 0.069	0.0001
Improvement ‐ moderate QoL	0.244 ± 0.645	0.0002
IPQ‐R identity		
Improvement ‐ low QoL	0.269 ± 0.152	0.079
Improvement ‐ moderate QoL	0.109 ± 0.155	0.48
IPQ‐R time (acute/chronic)		
Improvement ‐ low QoL	0.393 ± 0.112	0.0005
Improvement ‐ moderate QoL	0.285 ± 0.100	0.005
IPQ‐R personal control		
Improvement ‐ low QoL	−0.186 ± 0.085	0.029
Improvement ‐ moderate QoL	0.010 ± 0.081	0.90
IPQ‐R ‐ treatment control		
Improvement ‐ low QoL	−0.260 ± 0.116	0.025
Improvement ‐ moderate QoL	−0.118 ± 0.111	0.28
IPQ‐R ‐ illness coherence		
Improvement ‐ low QoL	−0.142 ± 0.064	0.029
Improvement ‐ moderate QoL	−0.105 ± 0.062	0.091
IPQ‐R‐ consequences		
Improvement ‐ low QoL	0.759 ± 0.158	< 0.0001
Improvement ‐ moderate QoL	0.404 ± 0.123	0.001
IPQ‐R—Time (cyclical)		
Improvement ‐ low QoL	0.140 ± 0.095	0.14
Improvement ‐ moderate QoL	0.087 ± 0.088	0.32
IPQ‐R‐ emotional representations		
Improvement ‐ low QoL	0.684 ± 0.158	< 0.0001
Improvement ‐ moderate QoL	0.473 ± 0.141	0.0009

Abbreviations: CEQ: Credibility and Expectancy Questionnaire; GAD: Generalised Anxiety Disorder Scale 7; IPQ: Illness Perception Questionnaire; PHQ: Patient Health Questionnaire 12; PHQ‐9: Patient Health Questionnaire 9; PSS: Perceived Stress Scale; VSI: Visceral Sensitivity Index.

### 3.3 Objective 2: Dynamic Relationships Over Time (CLPM)

**FIGURE 5:**
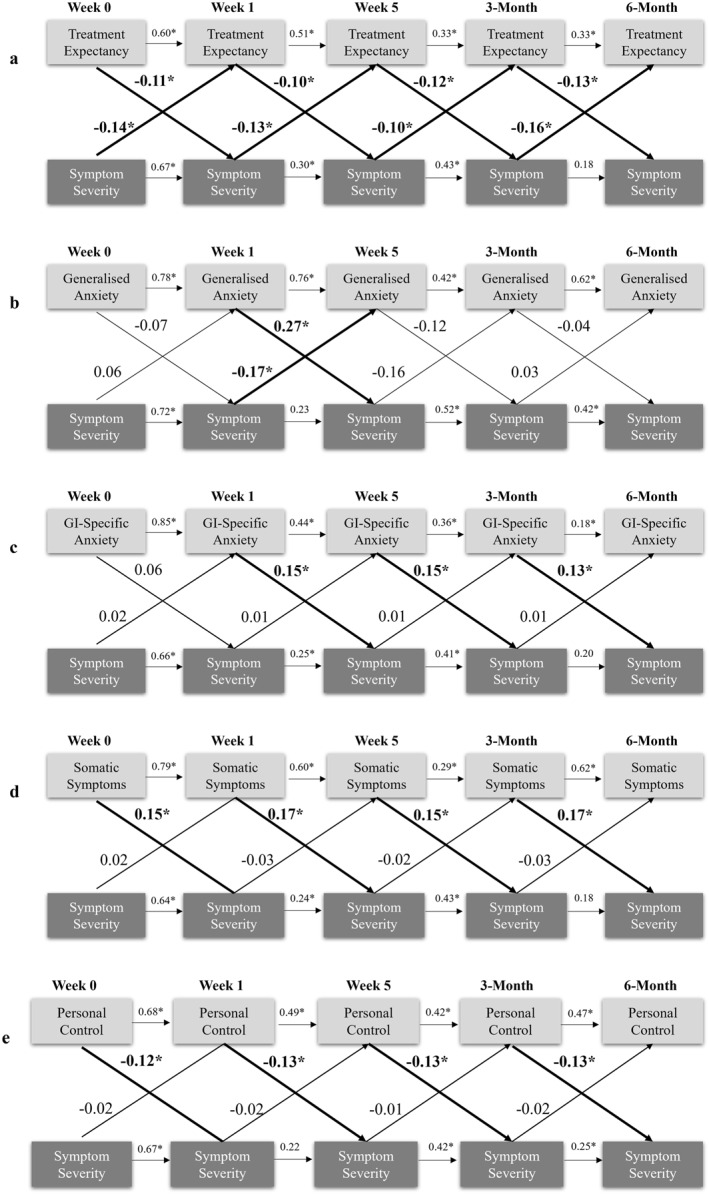
Cross‐lagged panel models showing directional relationships between gastro‐intestinal symptom severity (IBS‐SSS) and (a) treatment expectancy (CEQ), (b) generalised anxiety (GAD‐7), (c) gastrointestinal specific anxiety (VSI), (d) somatic symptom severity (PHQ‐12), and (e) personal control (IPQ‐R). Beta values represent standardised path coefficients. *Denotes significance (*p* < 0.05). CEQ: Credibility and Expectancy; GAD‐7: Generalised Anxiety Disorder Scale 7; GI: Gastrointestinal; IPQ‐R: Illness Perception Questionnaire‐Revised PHQ‐12: Patient Health Questionnaire 12; VSI: Visceral Sensitivity Index.

**FIGURE 6:**
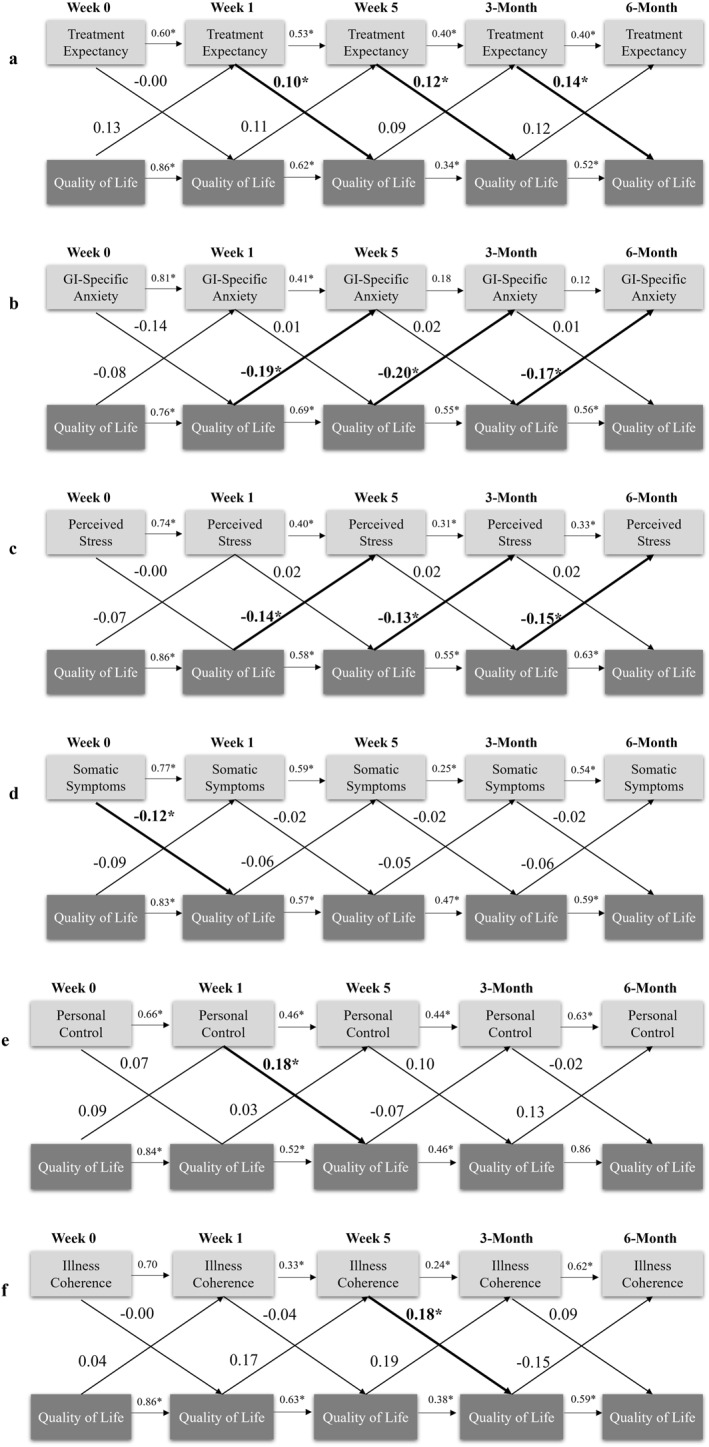
Cross‐lagged panel models showing directional relationships between quality of life (IBS‐QoL) and (a) treatment expectancy (CEQ), (b) gastrointestinal specific anxiety (VSI), (c) perceived stress (PSS), (d) somatic symptom severity (PHQ‐12), (e) personal control (IPQ‐R), and (f) illness coherence (IPQ‐R). Beta values represent standardised path coefficients. *Denotes significance (*p* < 0.05). CEQ: Credibility and Expectancy; GI: Gastrointestinal; IPQ‐R: Illness Perception Questionnaire‐Revised PSS: Perceived Stress Scale; PHQ‐12: Patient Health Questionnaire 12; VSI: Visceral Sensitivity Index.

Only predictor variables that significantly changed during the LFD were used in the CLPM. LMM analysis revealed general increases in treatment credibility and expectancy, and positive illness perceptions (personal control, illness coherence), and decreases in negative illness perceptions (IPQ‐R cyclical timeline and emotional representations), and psychological symptoms (PHQ‐9, GAD‐7, PHQ‐12, VSI, PSS; Supporting Information S2: Table 3) showing phase‐specific patterns of change over time (see Figure S1). Therefore, we explored the dynamic interrelationships between these variables and gastrointestinal symptom severity (Figure 5) and QoL (Figure 6) over time.

CLPM analyses identified directional associations between psychological factors and gastrointestinal symptom severity (Figure 5). After controlling for within‐variable stabilities over time and between‐variable correlations within time points, treatment credibility, depression, perceived stress, and illness coherence did not predict changes in gastrointestinal symptom severity or vice versa (i.e., cross‐lagged paths were insignificant). However, treatment expectancy and gastrointestinal symptoms showed bidirectional cross‐lagged effects: higher expectancy at time T consistently predicted greater reductions in gastrointestinal symptom severity at time *T* + 1, while lower symptom severity predicted greater increases in treatment expectancy at the subsequent time point (*p* < 0.05). Lower generalised anxiety at week 1 predicted symptom improvement at week 5 (*p* = 0.003), and vice versa (*p* = 0.031). Unidirectional effects emerged for GSA, somatic symptom severity, and personal control: lower levels at time T predicted greater symptom improvement at *T* + 1 (*p* 0 < 0.05), but not vice versa.

Cross‐lagged paths between QoL and psychological variables are shown in Figure 6. After controlling for within‐variable stabilities over time and between‐variable correlations within time points, treatment credibility, depression, and generalized anxiety did not predict changes in QoL or vice versa Higher treatment expectancy from week 1 onwards consistently predicted improved QoL at subsequent assessments (*p* < 0.010). Conversely, higher QoL predicted reduced GSA (*p* < 0.05) and perceived stress (*p* < 0.047) at the following assessments. Several additional unidirectional effects emerged: lower baseline somatic symptoms predicted higher QoL at week 1 (*p* = 0.020); higher personal control at week 1 predicted higher QoL at week 5 (*p* = 0.0003); and greater illness coherence at week 5 predicted improved QoL at week 13 (*p* = 0.009).

## 4 Discussion

This study provides the first comprehensive investigation of psychological predictors of response to a three‐phase LFD in adults with IBS. Our findings show consistent associations between higher treatment credibility and expectancy, more positive illness perceptions, lower baseline stress, depressive symptoms, GSA and somatic symptoms with greater symptom improvement trajectories. Cross‐lagged models also revealed bidirectional relationships between psychological factors and treatment outcomes. Notably, improvements in QoL preceded reductions in stress and GSA, suggesting that enhancing QoL may play a more central mechanistic role than previously assumed. These findings have important implications for patient selection and personalised IBS management.

Higher baseline treatment credibility and expectancy were associated with lower initial symptom severity and greater subsequent symptom improvement. This aligns with Expectancy Theory, which proposes that treatment expectations influence outcomes through psychological and neurobiological mechanisms [41]. Higher expectancy predicted both symptom reduction and improved QoL, while symptom improvement, in turn, reinforced expectancy. This pattern reflects a reciprocal relationship between expectations and symptoms, where early improvements may reinforce engagement and perceived treatment credibility. These findings highlight treatment expectancy as a modifiable factor to enhance dietary interventions. Clinicians can strengthen expectancy by providing clear education about the diet's rationale, evidence, and anticipated benefits.

Consistent with earlier studies, higher baseline anxiety, depressive and extraintestinal somatic symptoms, and perceived stress were associated with worse initial symptom severity and lower likelihood of improvement during the LFD [11]. Extending earlier work focused on general psychological distress, our analyses identified high GSA as a novel modifiable predictor of symptom persistence throughout the LFD. CLPM analysis revealed that decreases in GSA preceded reductions in gastrointestinal symptoms, whereas increases in QoL preceded improvements in psychological symptoms (stress and GSA) during the LFD. This challenges the assumption that reducing psychological symptoms is the primary pathway to improving QoL; instead, interventions primarily targeting QoL improvements may offer an alternative approach to breaking the symptom‐distress cycle. Lower GSA predicted better dietary response, whereas higher GSA has previously been shown to predict stronger responses to exposure‐based cognitive behavioural therapy (CBT) [42, 43]. Brief psychological screening using validated questionnaires, such as the VSI, CEQ, and IPQ‐R, could guide treatment allocation. At present, these instruments do not have established clinical cut‐offs to categorise ‘low’ or ‘high’ scores; however, defining clinically meaningful thresholds may improve their practical utility for treatment selection in clinical practice. Patients with low VSI scores, high treatment expectations, and strong personal control beliefs may benefit most from dietary interventions, whereas those with higher GSA, lower expectations, or maladaptive illness beliefs may benefit more from psychological therapy. For instance, exposure‐based CBT aims to reduce symptom‐related fear and avoidance through gradual exposure to feared GI sensations and situations, thereby increasing tolerance of GI discomfort and reducing avoidance behaviours [42, 43].

Our findings also indicated that more positive illness perceptions ‐ specifically lower illness identification, fewer perceived consequences, and lower emotional representations ‐ were associated with greater symptom and QoL improvement. This aligns with the Common‐Sense Model of self‐regulation, which posits that individuals who view their illness as less threatening and less disruptive are more likely to adopt adaptive coping strategies and adhere to treatment [44]. Targeting maladaptive illness beliefs, including through psychological interventions, may therefore enhance dietary adherence and improve outcomes in IBS [45]. Moreover, brief psychological screening is practical, low‐cost, and less resource‐intensive than biological profiling approaches (e.g., stool microbiome [46] or volatile organic compound analysis [47]), suggesting that pre‐treatment psychological screening is a feasible method to establish whether dietary or psychological interventions are indicated.

To our knowledge, this is the first study investigating psychological predictors of distinct symptom trajectories following a low FODMAP diet using LCGA. Several earlier studies have attempted to identify IBS patient subgroups based on combinations of symptom severity, QoL, psychological distress, and cognitive illness appraisal using latent class analysis. For example, Han et al. (2018) [48] reported four subgroups of individuals with IBS, largely differentiated by symptom severity, subtype, and QoL, with psychological distress and cognitive beliefs closely aligned with QoL. In contrast, Black et al. (2021, 2024) [49, 50] reported seven clusters in a large IBS cohort, driven not only by symptom type and severity but also by differences in burden of psychological distress, highlighting the importance of patient profiles. Extending beyond these primarily cross‐sectional findings, the present study examines the longitudinal interplay between psychological burden and symptoms and QoL trajectories across the low FODMAP diet. A further strength is the assessment of these relationships across all three phases of the diet, advancing prior research that has largely focused on the restriction phase alone. CLPMs enabled the identification of directional relationships between psychological factors and treatment outcomes over time, offering insight into potential mechanisms of change. Additional methodological strengths include blinding dietitians to psychological assessment and standardised intervention delivery by FODMAP‐trained (Monash University) dietitians.

Several limitations warrant consideration. The absence of a control group limits causal inference, as symptom changes over time cannot be solely attributed to the LFD and may reflect expectancy effects or regression to the mean or natural symptom fluctuations. However, this prospective design reflects routine clinical practice, and the analytic methods allow examination of trajectories and directional pathways that would not be feasible in a more constrained trial. Attrition increased across phases of the diet (63% by study completion), a pattern that is well‐recognised in real‐world LFD implementation, where engagement typically declines after the restriction phase. Although this reduces the generalisability of later‐phase findings, it does not compromise the early‐phase analyses where retention remained high. Moreover, because attrition is expected in multi‐phase dietary protocols, our use of LMM and FIML approaches ensures that all available data contribute to estimation, reducing the risk of biased inferences. While those with greater burden or lower early benefit may have been less likely to complete follow‐up, key psychological predictors were identified early, when the sample was largest and most representative. The predominantly female sample (89%) may restrict applicability, given potential sex differences in psychological responses to chronic GI symptoms [51], although this distribution reflects typical IBS clinic populations. Similarly, the single‐centre urban setting may limit generalisability to more diverse sociodemographic groups. Moreover, the predominantly female sample reflects the known gender distribution of IBS, but limits its applicability to males [1]. Participants also had facilitated access to low FODMAP foods and received intensive dietetic support, which may not reflect real‐world clinical settings and therefore may reduce generalisability. Although formal power calculations are not established for latent class growth analysis or cross‐lagged panel models, general guidance suggests larger sample sizes than that included here [38, 39], which would have also enabled examination of multivariate pre‐treatment psychological profiles rather than individual psychological factors separately. Future studies should therefore recruit larger and more diverse populations and embed engagement strategies across all phases of the diet to minimise expected drop‐off.

Our findings point to three key priorities for future research. First, a randomised controlled trial directly comparing the LFD and CBT, stratified by baseline GSA, is needed to test whether GSA reliably guides treatment selection. Second, factorial designs comparing single‐modality interventions (diet alone or psychological therapy alone) with combined approaches would clarify whether integrated treatments offer added benefit and help identify mediating mechanisms such as changes in anxiety or illness perceptions. Third, long‐term studies (12–24 months) comparing a range of IBS interventions including psychological therapies [52], stress management [53] and hypnotherapy [54] with dietary interventions should prioritise QoL as the primary endpoint and use frequent assessments to confirm the temporal pathways linking QoL and psychological change.

In conclusion, this study supports a precision approach to IBS management that aligns treatment with patient's psychological profiles, based on psychological predictors of response to a three‐phase LFD in IBS. The findings support a more personalised model of care in which treatment selection is informed by psychological profiles rather than symptom presentation alone. Identifying patients most likely to benefit from dietary versus psychological interventions may help clinicians optimise outcomes and avoid unnecessary or prolonged dietary restriction. Future work should validate these predictors in diverse clinical settings and establish evidence‐based thresholds that can be used to guide treatment decisions in clinical practice.

## Funding

Maaike Van Den Houte is a postdoctoral research fellow at the Research Foundation Flanders (FWO, 12A7U26N). Jessica Biesiekierski is supported by an Australian National Health and Medical Research Council Emerging Leadership fellowship (APP2025943).

## Conflicts of Interest

The authors declare no conflicts of interest.

## Supporting information


Supporting Information S1

Supporting Information S2

**Figure S1:** Phase‐specific patterns of change over time during the low FODMAP diet for treatment credibility and expectancy (CEQ), depressive and anxiety symptoms (GAD‐7), somatic symptoms (PHQ‐12), perceived stress (PSS), GI‐specific anxiety (VSI), and domains of illness perceptions (IPQ). Numbers represent least square means from mixed model analysis; error bars represent standard errors. During routine delivery of the LFD. Shading represents duration of each phase of the low FODMAP diet including Phase 1: Restriction, Phase 2: Reintroduction, and Phase 3: Personalisation.

## Data Availability

The data that support the findings of this study are available from the corresponding author upon reasonable request.
