# Supplementary material for: Psychological Factors Predict Response to a Low Fermentable Oligo‐, di‐, Monosaccharide and Polyol Dietary Intervention in Irritable Bowel Syndrome: A Prospective Cohort Study
Source: United European Gastroenterol J. 2026 Mar 24;14(3):e70204. doi: 10.1002/ueg2.70204 (PMC13140198; doi:10.1002/ueg2.70204)
Supplement: Supplementary file 1 — Supporting Information S1 [file UEG2-14-e70204-s003.docx]

**SUPPLEMENTARY MATERIAL**

**S1 – QUESTIONNAIRES**

*CEQ*

The Credibility and Expectancy Questionnaire (CEQ) was used to assess the participant’s beliefs about treatment credibility and expectations of the low fermentable oligosaccharide, di-saccharide, monosaccharide, and polyol (FODMAP) diet (LFD) for irritable bowel syndrome (IBS) management. The questionnaire contained two sets of questions related to credibility (set one; three questions) and expectations (set two; three questions) of treatment.^1^

Initially developed for trauma patients, the questionnaire was adapted to focus on the symptomatology of IBS, where the treatment offered was the LFD. Questions pertaining to set one (question two) and set two (questions one, two and three) had the word ‘trauma’ replaced with ‘IBS’. Sums for credibility and expectancy ranged from three to 27, where higher scores denote higher treatment credibility and expectancy.^1^

*PHQ-9*

The Patient Health Questionnaire 9 was used to assess symptoms of depression over the previous 14 days. The questionnaire assessed nine items over the previous 14 days and was scored by assigning score of 0 (none) to 3 (nearly every day) for each item. Higher scores denote increased symptoms of depression.^2^

*GAD-7*

The Generalised Anxiety Disorder Scale (GAD-7) was used to screen for generalised anxiety over the previous 14 days. The seven questions were scored from zero (score of 0 (none) to 3 (nearly every day). Higher scores indicate more severe anxiety symptoms.^3^

*PHQ-12*

The Patient Health Questionnaire was used to determine somatic symptom severity.^4^ Each question was scored with a response of ‘not bothered at all’ to ‘bothered a lot’. Higher scores indicate more severe somatic symptom severity. For analysis, the three gastrointestinal symptom items were removed to provide a more accurate indication of extraintestinal somatic symptoms, referred to as the PHQ-12.^5^

*PSS*The Perceived Stress Scale (PSS) was used to measure psychological stress by assessing thoughts and feelings over the previous four weeks.^6^ Participants responded to each question using the options never (zero points) to very often (four points). Higher scores indicate high perceived stress.

*VSI*

The Visceral Sensitivity Index (VSI) was used to measure fear, hypervigilance and anxiety-based responses to visceral sensations, known as gastrointestinal-specific anxiety.^7-8^ The questionnaire items are reverse-scored so that lower scores (zero) indicate lower gastrointestinal-specific anxiety and higher scores indicate severe gastrointestinal-specific anxiety.

*IPQ-R*

The Illness Perception Questionnaire-Revised (IPQ-R) was used to measure illness perceptions.^9^ The *first section* of the IPQ-R consists of the identity subscale, where participants identified if they have experienced a symptom and if they believed the symptom was associated with their IBS. There were 14 symptoms listed, such as fatigue, nausea, and upset stomach, and the total number of ‘yes’ responses relating to being associated with IBS comprised the score for the identity domain. The *second section* of the IPQ-R consists of 38 questions on illness beliefs that inform seven subscales: acute/chronic timeline, illness consequences, cyclical timeline, personal control, treatment control, illness coherence, and emotional representation. This section was scored using a five-point Likert scale from one (strongly disagree) to five (strongly agree). The *third* section was not used in this study.

2.5 Outcomes

*IBS-QoL*

The IBS Quality of Life (IBS-QoL) scale was used to quantify disease-specific QoL. The questionnaire contains 34 items focused on the previous 30 days, which are scored on a one (not at all) to five (a great deal) Likert scale.^10-11^All items were reverse scored, then transformed to yield a final score ranging from zero to 100 using the following formula, such that a higher score on the IBS-QoL indicates a better QoL: *Transformed score = sum of all items – lowest possible score / possible score range X 100*

*IBS-SSS*Symptom severity was assessed using the IBS Symptom Severity Score (IBS-SSS).^12^ The questionnaire contains seven items; two questions identify if the participant was suffering symptoms at the time of completion with a ‘yes’ or ‘no’ response option, one questions requires identification of the number of days that pain was felt in the previous 10-day period and the four remaining items were scored on a 100mm visual analogue scale from zero (no pain or distention, or very satisfied with bowel habits) to 100 (very severe pain or distention, or very unhappy with bowel habits). To obtain a total IBS-SSS score, the question identifying the number of days pain was felt in the previous 10-day period was multiplied by 10 (i.e., seven days x 10 = 70), which was added to the scores from the four visual analogue scales. Total symptom severity scores can range from zero to 500, where scores can be further classified mild (75-<175), moderate (>175 - <300) and severe (>300).

**References**

1. G.J. Devilly and T.D. Borkovec, “Psychometric Properties of the Credibility/Expectancy Questionnaire,” *Journal of Behavior Therapy and Experimental Psychiatry* 31, no.2 (2000): 73-86, doi: 10.1016/S0005-7916(00)00012-4.
2. K. Kroenke, R.L. Spitzer, J.B.W. Williams. “The PHQ-9: Validity of a Brief Depression Severity Measure,” *Journal of General Internal Medicine* 16, no. 9 (2001): 606-613, doi: 10.1046/j.1525-1497.2001.016009606.x.
3. L Bernd, D. Oliver, M. Stefanie et al., “Validation and Standardization of the Generalized Anxiety Disorder Screener (GAD-7) in the General Population,” *Medical Care* 46, no.3 (2008): 266-74, doi: 10.1097/MLR.0b013e318160d093.
4. Kroenke K, Spitzer RL, Williams JBW. The PHQ-15: validity of a new measure for evaluating the severity of somatic symptoms. Psychosom Med. 2002;64:258-66.
5. R.C. Spiller, D.J. Humes, E. Campbell et al., “The Patient Health Questionnaire 12 Somatic Symptom scale as a Predictor of Symptom Severity and Consulting Behaviour in Patients with Irritable Bowel Syndrome and Symptomatic Diverticular Disease,”  *Alimentary Pharmacology & Therapeutics* 32, no. 6 (2010): 811-20, doi: 10.1111/j.1365-2036.2010.04402.x.
6. S. Cohen, T. Kamarck, R and Mermelstein., “A Global Measure of Perceived Stress,” *Journal of Health and Social Behavior* 24, no.4 (1983): 385-96, doi: 10.2307/2136404.
7. J.S. Labus, R. Bolus, L. Chang et al., “The Visceral Sensitivity Index: Development and Validation of a Gastrointestinal Symptom‐Specific Anxiety Scale,” *Alimentary Pharmacology & Therapeutics* 20, no.1. (2004): 89-97, doi: 10.1111/j.1365-2036.2004.02007.x.
8. J.S. Labus, E.A. Mayer, L. Chang et al., “The Central Role of Gastrointestinal-Specific Anxiety in Irritable Bowel Syndrome: Further Validation of the Visceral Sensitivity Index,” *Psychosomatic Medicine* 69, no.1 (2007): 89-98, doi: 10.1097/PSY.0b013e31802e2f24
9. R. Moss-Morris, J. Weinman, K. Petrie et al., “The Revised Illness Perception Questionnaire (IPQ-R),” *Psychology & Health* 17, no.1 (2002): 1-16, doi: 10.1080/08870440290001494.
10. D.L. Patrick, D.A. Drossman, I.O. Frederick et al., “Quality of Life in Persons with Irritable Bowel Syndrome: Development and Validation of a New Measure,” *Digestive Diseases and Sciences* 43, no.2 (1998): 400-11, doi: 10.1023/a:1018831127942
11. D.A. Drossman, D.L. Patrick and W.E.Whitehead et al., “Further Validation of the IBS-QOL: a Disease-Specific Quality-of-life Questionnaire,” *The American Journal of Gastroenterology* 95, no. 4 (2000): 999-1007, doi: 10.1016/S0002-9270(00)00733-4.
12. C.Y. Francis, J. Morris and P.J. Whorwell, “The Irritable Bowel Severity Scoring System: a Simple Method of Monitoring Irritable Bowel Syndrome and its Progress,” *Alimentary Pharmacology & Therapeutics* 11, no.2 (1997): 395-402, doi: 10.1046/j.1365-2036.1997.142318000.x.

**S2 – THE LOW FODMAP DIET**

*Phase 1 FODMAP Restriction (weeks 1-5)*

Appointment one had a duration of 60 minutes and was conducted in person or online. The dietitian collected a detailed history of symptoms and an assessment of FODMAP intake to help inform dietary changes. Participants were provided with education on the mechanisms of fermentable carbohydrates in IBS to rationalize the restriction of multiple FODMAP constituents. Participants were provided with educational resources that detailed high and low-FODMAP-containing foods, suggested swaps based on their dietary intake and websites with suitable recipes. The participants were encouraged to reduce high FODMAP foods by reducing the portion consumed or swapping foods for low FODMAP alternatives to reduce the total FODMAP intake during Phase 1.

*Phase 2 FODMAP Reintroduction (weeks 6-13)*

Forty-five minutes was allocated to the second appointment. Another dietary assessment was completed to ascertain that the participant had reduced their FODMAP intake enough to allow for FODMAP reintroduction to occur. If participants had ongoing symptoms and high FODMAP foods were identified, they were offered an additional one to two weeks to elicit a further symptom response. The dietitian provided education on the reintroduction protocol, which included written instructions on challenging each of the FODMAP groups (including food types to be used, portion sizes, and frequency of consumption). The reintroduction protocol was informed by the published data.^1^ A three-day washout period between each reintroduction was encouraged, and participants were instructed to maintain a low FODMAP for the duration of Phase 2 unless challenging a FODMAP-containing food. Participants could choose the order in which they rechallenged foods and were provided with a template to record food and symptom responses. Participants were encouraged to complete the reintroduction period over eight weeks but were permitted an additional two weeks to finalise any unattempted challenges. Participants who felt they did not adequately respond to Phase 1, irrespective of IBS-SSS scores, did not proceed to Phase 2.

*Phase 3 FODMAP Personalisation (week 13 onwards)*

Thirty minutes was allocated to the final appointment, which was used to interpret FODMAP challenges and personalise the participant's diet. Participants were provided with education around appropriate thresholds for each FODMAP, with guidance on suitable foods and portions to consume going forward. If there was uncertainty around a challenge, participants were encouraged to maintain a low FODMAP diet so that the FODMAP group could be rechallenged before moving into the personalisation phase (3 months to 6 months). Foods that did not exacerbate symptoms were encouraged to be reintroduced into the diet.

**References**

1. C.J. Tuck, and J. Barrett,“Re‐Challenging FODMAPs: the Low FODMAP Diet Phase Two,” *Journal of Gastroenterology and Hepatology* 31 (2017):11-15, doi: 10.1111/jgh.13687.

**S3 – STATISTICAL ANALYSIS**

*Objective 1: cognitive factors and psychological symptoms as predictors of response to the low FODMAP diet*

The optimal clustering solution was estimated by adding classes to the model and using the Bayesian Information Criteria (BIC) to determine the best statistical fit.^1^ Once the response trajectories were determined, the significance of between-class differences was tested for the intercept, linear, quadratic, and cubic slopes using one-way ANOVAs and post-hoc t-tests for all pairwise differences between classes. Baseline scores for predictor variables (treatment credibility, treatment expectancy, illness perceptions, general anxiety, gastrointestinal specific anxiety, depressive, and somatic symptom severity were individually added to the model as risk factors and tested against a reference class to determine their contribution to the likelihood of being allocated to each of the response trajectories. LCGA was implemented using SAS proc traj.

*Objective 2: dynamic relationship between cognitive factors and psychological symptoms on the one hand, and gastrointestinal symptoms and QoL on the other hand, over time*

Cross-lagged paths within the CLPM determined if the “predictor” variable scores (X) at each time point T could predict a change in the “outcome” variable (Y) at time T+1 and vice versa, while controlling for within-variable stabilities over time and within-timepoint covariances between X and Y (**Figure 2**). Standardised path coefficients (β) are reported for paths between variables and time points. For each combination of variables, three models were run: one with equality constraints (assuming the strength of the cross-lagged paths is consistent across all time points), one with equality constraints released, and one with partial equality constraints (assuming the strength of cross-lagged paths is consistent over time except for T=0 to T=1). The shorter duration between these two time points may result in a weaker path relationship due to the minimal change that occurred during that time. **Figure 2** displays a cross-lagged panel model, showing the predictive pathways and whether equality constraints were applied.

The fit of each model was assessed using several fit indices with their respective cut-offs: Chi² p-value (> 0.05), root mean square error of approximation (RMSEA) (< 0.05), and the Bentler comparative fit index (BCFI) (> 0.90). The CLPM that produced the best fit was further enhanced by adding sensible stabilities that crossed multiple time points, as per the suggested modification indices. This process continued until no better fit could be achieved. Only CLPMs with significant cross-lagged pathways are reported in detail. Cross-sectional pathways and added stabilities are omitted from the figures for clarity.

**References:**

1. A.A. Neath and J.E. Cavanaugh, “The Bayesian information criterion: background, derivation, and applications,” *WIREs Computational Statistics* 4 (2012):199-203, doi:10.1002/wics.199
