# Supplementary material for: Psychological Factors Predict Response to a Low Fermentable Oligo‐, di‐, Monosaccharide and Polyol Dietary Intervention in Irritable Bowel Syndrome: A Prospective Cohort Study
Source: United European Gastroenterol J. 2026 Mar 24;14(3):e70204. doi: 10.1002/ueg2.70204 (PMC13140198; doi:10.1002/ueg2.70204)
Supplement: Supplementary file 2 — Supporting Information S2 [file UEG2-14-e70204-s002.docx]

***Supplementary Table 1:*** *Intercepts and linear, quadratic, and cubic slopes for each symptom (IBS-SSS) class trajectory of the latent class growth analysis. P-values indicate differences from zero. Estimates in the same row with the same superscript are not significantly different from each other.*

|  | **Improvement - low symptom severity**  **(22.5%)** | | | **Improvement - moderate symptom severity (56.2%)** | | | **Non-improvement**  **(21.3%)** | | |
| --- | --- | --- | --- | --- | --- | --- | --- | --- | --- |
|  | **Estimate** | **SE** | **p** | **Estimate** | **SE** | **p** | **Estimate** | **SE** | **p** |
| **Intercept** | 196.3^a^ | 10.1 | < 0.0001 | 284.2^b^ | 7.61 | < 0.0001 | 352.5^c^ | 8.7 | < 0.0001 |
| **Linear slope** | -20.0^a^ | 6.57 | 0.002 | -19.5^a^ | 4.09 | < 0.0001 | -2.0^b^ | 0.8 | 0.013 |
| **Quadratic slope** | 1.72^a^ | 0.76 | 0.024 | 1.25^a^ | 0.48 | 0.010 |  |  |  |
| **Cubic slope** | -0.041^a^ | 0.021 | 0.046 | -0.025^a^ | 0.013 | 0.064 |  |  |  |

***Supplementary Table 2:*** *Intercepts and linear, quadratic, and cubic slopes for each quality of life (IBS-QOL) class trajectory of the latent class growth analysis. P-values indicate differences from zero. Estimates in the same row with the same superscript are not significantly different from each other.*

|  | **Improvement - low QoL**  **(22.5%)** | | | **Improvement - moderate QoL**  **(56.2%)** | | | **High QoL**  **(21.3%)** | | |
| --- | --- | --- | --- | --- | --- | --- | --- | --- | --- |
|  | **Estimate** | **SE** | **p** | **Estimate** | **SE** | **p** | **Estimate** | **SE** | **p** |
| **Intercept** | 38.4 | 1.44 | < 0.0001 | 63.3 | 1.53 | < 0.0001 | 87.8 | 1.98 | < 0.0001 |
| **Linear slope** | 1.48 | 0.41 | 0.0004 | 3.17 | 0.80 | 0.0001 |  |  |  |
| **Quadratic slope** | -0.042 | 0.017 | 0.014 | -0.25 | 0.095 | 0.008 |  |  |  |
| **Cubic slope** |  |  |  | 0.006 | 0.003 | 0.024 |  |  |  |

***Supplementary Table 3:*** *Linear mixed model trajectories testing the effect of time on each questionnaire.*

|  | | | | |
| --- | --- | --- | --- | --- |
| **Variable** | **Intercept ß ± SE** | **Linear ß ± SE** | **Quadratic ß ± SE** | **Cubic ß ± SE** |
| **CEQ- Credibility** | 19.4 ± 0.3*** | 0.732 ± 0.162*** | -0.063 ± 0.019** | 0.0015 ± 0.0005** |
| **CEQ- Expectancy** | 19.8 ± 0.3*** | 0.336 ± 0.148* | -0.035 ± 0.017* | 0.0010 ± 0.0005* |
| **PHQ-9** | 7.0 ± 0.4*** | -0.856 ± 0.116*** | 0.072 ± 0.014*** | -0.0017 ± 0.0004*** |
| **GAD-7** | 5.9 ± 0.4*** | -0.567 ± 0.124*** | 0.053 ± 0.015** | -0.0013 ± 0.0004** |
| **PHQ-12** | 8.1 ± 0.3*** | -0.802 ± 0.102*** | 0.074 ± 0.012*** | -0.0018 ± 0.0003*** |
| **PSS** | 16.5 ± 0.6*** | -0.450 ± 0.213* | 0.024 ± 0.025 | -0.0004 ± 0.0007 |
| **VSI** | 49.1 ± 1.4*** | -2.974 ± 0.454*** | 0.238 ± 0.054*** | -0.0055 ± 0.0015** |
| **IPQ-R Identity** | 4.8 ± 0.2*** | -0.002 ± 0.077 | -0.007 ± 0.009 | 0.0002 ± 0.0003 |
| **IPQ-R Time (acute/chronic)** | 23.8 ± 0.4*** | -0.224 ± 0.146 | 0.019 ± 0.017 | -0.0004 ± 0.0005 |
| **IPQ-R Consequences** | 18.6 ± 0.4*** | -0.189 ± 0.126 | 0.020 ± 0.015 | -0.0005 ± 0.0004 |
| **IPQ-R Personal Control** | 22.8 ± 0.4*** | 0.449 ± 0.133*** | -0.044 ± 0.016** | 0.0011 ± 0.0004* |
| **IPQ-R Treatment Control** | 18.4 ± 0.3*** | 0.125 ± 0.111 | -0.015 ± 0.013 | 0.0004 ± 0.0004 |
| **IPQ-R Illness Coherence** | 14.8 ± 0.5*** | 1.299 ± 0.173*** | -0.119 ± 0.020*** | 0.0029 ± 0.0006*** |
| **IPQ-R Time (cyclical)** | 14.9 ± 0.3*** | -0.276 ± 0.120* | 0.022 ± 0.014 | -0.0005 ± 0.0004 |
| **IPQ-R Emotional Representations** | 19.2 ± 0.4*** | -0.403 ± 0.143** | 0.027 ± 0.017 | -0.0006 ± 0.0005 |

*CEQ: Credibility and Expectancy Questionnaire; PHQ-9: Patient Health Questionnaire-9; GAD-7: Generalized Anxiety Disorder Scale -7; PHQ-12: Patient Health Questionnaire-12; PSS: Perceived Stress Scale; VSI: Visceral Sensitivity Index; IBS-BRQ: Irritable Bowel Syndrome Behavioural Response Questionnaire; IBS-SSS: Irritable Bowel Syndrome Symptom Severity Score; IBS QoL: Irritable Bowel Syndrome Quality of Life; IPQ-R: Illness Perception Questionnaire-Revised; SE: Standard Error. ***p < .0001; **p < .01; *p < .05*
