# Supplementary figures and images for: Psychological Factors Predict Response to a Low Fermentable Oligo‐, di‐, Monosaccharide and Polyol Dietary Intervention in Irritable Bowel Syndrome: A Prospective Cohort Study
Source: United European Gastroenterol J. 2026 Mar 24;14(3):e70204. doi: 10.1002/ueg2.70204 (PMC13140198; doi:10.1002/ueg2.70204)

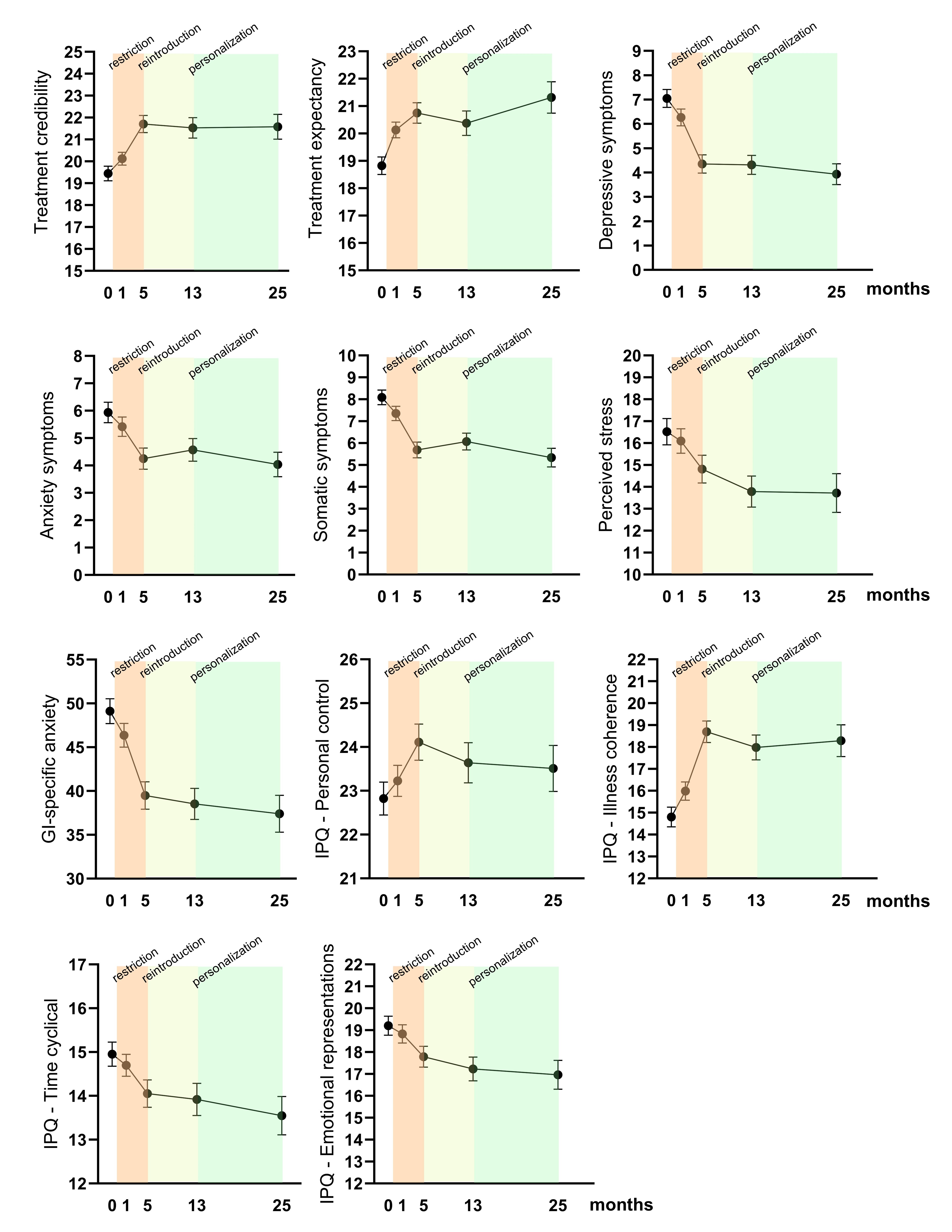

Supplement: Supplementary file 3 — Figure S1: Phase‐specific patterns of change over time during the low FODMAP diet for treatment credibility and expectancy (CEQ), depressive and anxiety symptoms (GAD‐7), somatic symptoms (PHQ‐12), perceived stress (PSS), GI‐specific anxiety (VSI), and domains of illness perceptions (IPQ). Numbers represent least square means from mixed model analysis; error bars represent standard errors. During routine delivery of the LFD. Shading represents duration of each phase of the low FODMAP diet including Phase 1: Restriction, Phase 2: Reintroduction, and Phase 3: Personalisation. [file UEG2-14-e70204-s001.jpg]
